# Survival effect of pre-RT PET-CT on cervical cancer: Image-guided intensity-modulated radiation therapy era

**DOI:** 10.3389/fonc.2023.1012491

**Published:** 2023-01-31

**Authors:** Chih-Hsiung Su, Wan-Ming Chen, Mingchih Chen, Ben-Chang Shia, Szu-Yuan Wu

**Affiliations:** ^1^ Department of Accounting Information, Chihlee University of Technology, Taipei, Taiwan; ^2^ Graduate Institute of Business Administration, College of Management, Fu Jen Catholic University, Taipei, Taiwan; ^3^ Artificial Intelligence Development Center, Fu Jen Catholic University, Taipei, Taiwan; ^4^ Department of Food Nutrition and Health Biotechnology, College of Medical and Health Science, Asia University, Taichung, Taiwan; ^5^ Division of Radiation Oncology, Lo-Hsu Medical Foundation, Lotung Poh-Ai Hospital, Yilan, Taiwan; ^6^ Big Data Center, Lo-Hsu Medical Foundation, Lotung Poh-Ai Hospital, Yilan, Taiwan; ^7^ Department of Healthcare Administration, College of Medical and Health Science, Asia University, Taichung, Taiwan; ^8^ Cancer Center, Lo-Hsu Medical Foundation, Lotung Poh-Ai Hospital, Yilan, Taiwan; ^9^ Centers for Regional Anesthesia and Pain Medicine, Taipei Municipal Wan Fang Hospital, Taipei Medical University, Taipei, Taiwan; ^10^ Department of Management, College of Management, Fo Guang University, Yilan, Taiwan

**Keywords:** IG-IMRT, ^18^FDG-PET–CT, cervical carcinoma, survival, clinical stages

## Abstract

**Condensed abstract:**

No large-scale, well-designed randomized study with a long-term follow-up has evaluated the survival effect of pretreatment 18-fluorodeoxyglucose positron emission tomography–computed tomography (^18^FDG-PET–CT) on patients with stage IB–IVA cervical cancer receiving image-guided intensity-modulated radiation therapy (IG-IMRT). This is the first head-to-head propensity score–matched, nationwide population-based cohort study evaluating this survival effect. The results revealed that pretreatment ^18^FDG-PET–CT might be associated with longer survival in patients with stage IB–IVA cervical cancer receiving radiotherapy or concurrent chemoradiotherapy, especially in the IG-IMRT era.

**Purpose:**

No large-scale, well-designed randomized study with a long-term follow-up has evaluated the survival effect of pretreatment 18-fluorodeoxyglucose positron emission tomography–computed tomography (^18^FDG-PET–CT) on patients with stage IB–IVA cervical cancer receiving image-guided intensity-modulated radiation therapy (IG-IMRT). Therefore, in this propensity score–matched, population-based cohort study, we investigated these survival effects.

**Patients and methods:**

We included 4167 patients with stage IB–IVA cervical cancer receiving radiotherapy (RT) or concurrent chemoradiotherapy (CCRT) through the IG-IMRT technique. The patients were categorized into two 1:2 propensity score–matched groups depending on whether they underwent pretreatment ^18^FDG-PET–CT, and their outcomes were compared.

**Results:**

We included 2778 and 1389 patients with cervical cancer in the nonpretreatment and pretreatment PET–CT groups, respectively. Univariable and multivariable analyses revealed an association between pretreatment PET–CT and improved survival in the patients (in the adjusted model, the adjusted hazard ratio [aHR] was 0.88; 95% confidence interval [CI], 0.80–0.97: *P* = 0.010). Regardless of the cancer stage (early or advanced), pretreatment PET–CT was significantly superior to nonpretreatment PET–CT in terms of all-cause death (aHR, 0.78; 95% CI, 0.60–0.92; *P* = 0.013 and aHR, 0.90; 95% CI, 0.81–0.99; *P* = 0.039 for the early [IB–IIA] and advanced stages [IIB–IVA], respectively).

**Conclusions:**

Pretreatment ^18^FDG-PET–CT might be associated with longer survival in patients with stage IB–IVA cervical cancer receiving RT or CCRT, especially in the era of IG-IMRT.

## Introduction

Although cervical cancer is the fourth most common cancer in women globally, the number of cervical cancer cases has continuously declined in regions that have implemented screening programs (1). However, in resource-poor areas with no well-established screening programs, the incidence and mortality rates of cervical cancer remain disproportionately high. (1) Cervical cancer is the leading cause of cancer deaths in 42 countries, with the majority of cases being reported from sub-Saharan Africa and Southeast Asia (1). Compared with other gynecological cancers, cervical cancer more commonly affects younger women, with a mean age at diagnosis of 49 years.

Following the diagnosis of cervical cancer, a pretreatment staging evaluation is performed in all women to determine the treatment approach, which can then be stratified on the basis of whether the disease is early or locally advanced at presentation (2). Accurate cancer staging is the most vital for planning optimal treatments and thus for optimal survival (3). For patients with early-stage cervical cancer, surgery alone without adjuvant therapy or radiotherapy (RT) alone might be suitable based on the National Comprehensive Cancer Network (NCCN) guidelines. However, for patients with advanced-stage cervical cancer without metastasis, concurrent chemoradiotherapy (CCRT) might be necessary (2).

Prior to RT or CCRT, whole-body 18-fluorodeoxyglucose (^18^FDG) positron emission tomography (PET)–computed tomography (CT) is conducted to evaluate the extent of the disease, with a particular focus on lymph node metastases, for obtaining information necessary to design RT fields (4). Compared with CT alone, PET–CT exhibits higher sensitivity for the detection of abdominal lymph node metastasis, a feature that affects the RT fields and estimates of patient prognosis (5, 6). However, no long-term follow-up study with an adequate sample size has evaluated the benefits of pretreatment PET–CT, which offers the most accurate imaging results for lymph node metastasis (7, 8), and its contribution to overall survival (OS) in patients with cervical cancer receiving RT or CCRT, especially in the era of image-guided intensity-modulated radiation therapy (IG-IMRT).

## Patients and methods

### Study design and patient data source

This retrospective study was conducted using data from the Health and Welfare Data Center (HWDC), established by Taiwan’s Ministry of Health and Welfare. Data gathered by the Taiwanese government from various sources are consolidated by HWDC, deidentified, and made available for research purposes based on case-by-case approval. We particularly used data from the Taiwan Cancer Registry (TCR), which includes the detailed staging and treatment information of patients with cancer; the Cause of Death database, which lists all death certificates issued in Taiwan (9–11); and the National Health Insurance Research Database (NHIRD), which contains the claims data of all National Health Insurance (NHI)-reimbursed examinations, medications, and treatments. Absence of the evidence of death cannot be considered the evidence of life because all death certificates issued are government system–specific judgments. Without a death certificate, property inheritance, abandonment of inheritance to the court, burial, or cremation cannot be performed in Taiwan. The NHI program has been implemented since 1995 and covers more than 99% of Taiwan’s population. Since July 2004, the NHI has been reimbursing ^18^F-FDG-PET performed for the initial staging of cervical cancer when optimal staging was unachievable through conventional imaging modalities. All PET-CT scans were reviewed and reported by a professional nuclear medicine physician in the study. All HWDC databases are linked through a common but anonymized identifier to ensure privacy. The requirement for informed consent was waived because of the retrospective nature of the study and the use of deidentified data.

### Inclusion and exclusion criteria

Patients were enrolled if they were diagnosed as having cervical cancer on the basis of pathological reports between January 1, 2008, and December 31, 2018, were aged ≥20 years, and had an American Joint Committee on Cancer (AJCC) clinical stage of IB–IVA based on the eighth edition. The diagnoses were confirmed using pathological data, and patients who were newly diagnosed as having cervical squamous cell carcinoma or adenocarcinoma were confirmed to have no other cancer. Patients with distant metastasis, cancer of unknown pathologic type, missing sex data, age <20 years, or unclear staging were excluded. In addition, patients were excluded if they did not receive RT within 3 months of diagnosis, RT involving contemporary IG-IMRT techniques, or a weekly platinum-based chemotherapy regimen during RT. Moreover, we excluded patients who received only sequential chemotherapy and RT. In this study, pelvic RT comprised external beam radiotherapy (EBRT) delivered once daily for 25–28 days for a total median dose of 50.4 Gy with IG-IMRT. Radiation oncologists in Taiwan prescribed total intracavity brachytherapy (ICBT) with a high-dose rate system, with a median dose of 25 Gy in five fractions when administered with or without concurrent chemotherapy. IMRT, a highly conformal EBRT technique, and its iteration—image-guided volumetric modulated arc therapy—were allowed in the study.

### Covariates and outcome definition

Data regarding age, histology, AJCC clinical stages, treatments (RT alone or CCRT), tumor differentiation, EBRT cumulative dose, platinum cumulative dose, ICBT cumulative dose, Charlson comorbidity index (CCI) scores, diagnosis year, and hospital levels (medical or nonmedical center) at the last follow-up were extracted from the TCR. Age was considered a continuous variable. All patients with nonmetastatic cervical cancer underwent RT alone or definitive CCRT in accordance with NCCN guidelines. The date of RT initiation was the index date.

From the NHIRD, we identified patients who underwent ^18^F-FDG PET–CT within 0–90 days before the index date. Only patients with a record of ^18^F-FDG-PET–CT were considered to have undergone pretreatment PET–CT. All patients in the control group (nonpretreatment ^18^FDG-PET–CT) received pelvic magnetic resonance imaging (MRI) with contrast to determine the local disease extent and chest/abdominal/pelvis CT for at least metastatic staging. The primary outcome of interest was all-cause death, which was calculated from the initial date to the date of death. Information on OS was obtained from the Cause of Death database. Patients with no death records were considered alive and censored on the last day of the database record (December 31, 2019).

### Propensity score matching

After adjustment for confounders, we performed Cox proportional hazards regression to model time from the index date to all-cause death for patients with cervical cancer who underwent RT or CCRT. We used propensity score matching (PSM) to reduce confounding factors, thus controlling these factors and elucidating the directionality of the survival effect of pretreatment PET–CT. The following matching variables were employed: age, histology, differentiation, AJCC clinical stages, treatments, cumulative EBRT dose, cumulative platinum dose, cumulative ICBT dose, CCI scores, diagnosis year, and hospital levels. However, because a residual imbalance was noted in covariates, (12) multivariable Cox proportional regression models were used. For the CCI score calculation, comorbidities were determined according to the *International Classification of Diseases, Ninth or Tenth Revision, Clinical Modification (ICD-9-CM* or *ICD-10-CM*) codes in the main diagnosis of inpatient records or those in outpatient records if the number of outpatient visits was ≥2 within 1 year. Comorbidities with onset 12 months before the index date were recorded. We matched the cohorts at a ratio of 2:1 by using a greedy matching method, and covariates were matched with a propensity score within a caliper of 0.2. (13).

### Statistical analysis

Continuous data are presented as the mean ± standard deviation or median (interquartile range), as applicable, and categorical data as the number and percentage. The distribution of patient characteristics was compared using the χ^2^ test for categorical variables and the independent *t* test or Kruskal–Wallis test for continuous variables.

Survival curves were generated using the Kaplan–Meier and compared using the log-rank test. In addition, the Kaplan–Meier curves of overall survival (OS) for different stages of cervical cancer with or without pretreatment PET–CT were compared using the log-rank test. Cox proportional hazards models were used to estimate the hazard ratio (HR) and 95% confidence interval (CI) and to determine the effects of covariates on OS. Stratified analysis was performed to investigate the effect of pretreatment PET–CT on various AJCC clinical stages (IB–IIA and III–IVA) and on OS across various subgroups. All statistical analyses were performed using SAS (version 9.4; SAS Institute). A two-sided *P* value of <0.05 was considered significant.

### Ethical approval

The study protocols were reviewed and approved by the Institutional Review Board of Tzu-Chi Medical Foundation (IRB109-015-B).

## Results

### Patient characteristics

A total of 4167 patients with cervical cancer met the inclusion criteria after PSM (**Table 1**). Of them, 2778 and 1389 patients who underwent RT or CCRT were included in the nonpretreatment and pretreatment PET–CT groups, respectively. All covariates were balanced between the groups after PSM (**Table 1**). The crude mean follow-up periods and crude mortality rates of the pretreatment and nonpretreatment PET–CT groups were 4.72 and 4.70 years and 45.64% and 51.08% (*P* < 0.001), respectively.

**Table 1:** Clinicodemographic characteristics of patients with cervical cancer with and without pretreatment PET–CT scan before RT or CCRT (after propensity score matching).

	Nonpretreatment PET–CT		Pretreatment PET–CT		*P* value	SMD
	N = 2778		N = 1389			
	N	%	N	%		
**Age** (mean ± SD)	61.81 ± 14.15		61.65 ± 13.95		0.224	0.051
Age, median (IQR), y	60.20 (51.00, 71.00)		60.00 (50.00, 70.00)		0.311	
Age					0.187	0.010
Age ≤ 40 y	612	22.03%	309	22.25%		
40 y < Age ≤ 50 y	833	29.99%	432	31.10%		
50 y < Age ≤ 60 y	667	24.01%	289	20.81%		
Age > 60 y	666	23.97%	359	25.85%		
**Years of diagnosis**					0.188	0.067
2008–2010	532	19.15%	241	17.35%		
2011–2014	1086	39.09%	531	38.23%		
2015–2018	1160	41.76%	617	44.42%		
**CCI scores** (mean ± SD)	0.41 ± 1.00		0.41 ± 0.88		0.709	0.017
CCI scores					0.371	0.016
0	2093	75%	1064	76.60%		
1	685	25%	325	23.40%		
**AJCC stages**	2778		1389		0.279	0.083
IB	437	15.73%	190	13.68%		
IIA	207	7.45%	90	6.48%		
IIB	891	32.07%	446	32.11%		
III	671	24.15%	346	24.91%		
IVA	566	20.37%	313	22.53%		
Missing	6	0.22%	4	0.29%		
**Differentiation**					0.220	0.072
I (well-differentiated)	84	3.02%	42	3.02%		
II (moderately differentiated)	1532	55.15%	814	58.60%		
III (poorly differentiated)	1089	39.20%	467	33.62%		
IV (undifferentiated)	73	2.63%	66	2.38%		
**Treatments**					0.861	0.022
CCRT	2111	75.99%	1055	75.95%		
RT alone	667	24.01%	334	24.05%		
**EBRT cumulative dose**, Gy						
Mean (SD)	50.40 ± 18.70		50.40 ± 19.24		0.999	0.1000
Median (Q1–Q3)	50.40 (39.33, 60.00)		50.40 (46.00, 60.00)		0.999	
**Chemotherapy,** Platinum cumulative dose, mg						
Mean (SD)	632.33 ± 593.38		639.02 ± 577.10		0.753	0.011
Median (Q1–Q3)	500.00 (350.00, 600.0)		500.00 (350.00, 650.0)		0.224	
**Brachytherapy** dose, Gy						
Mean (SD)	24.68 ± 6.57		24.14 ± 6.55		0.717	0.014
Median (Q1–Q3)	25.00 (20.00, 30.00)		2500.00 (20.00, 30.00)		0.999	
**Histological type**					0.974	0.002
Adenocarcinoma	298	10.73%	150	10.80%		
Squamous cell carcinoma	2480	89.27%	1239	89.20%		
**Medical centers**					0.426	0.019
Nonmedical centers	1517	10.73%	745	53.64%		
Medical centers	1261	45.39%	644	46.36%		
Mean (SD) follow-up (y)	4.70 ± 2.83		4.72 ± 2.64		0.828	
Median (IQR) follow-up (y)	4.04 (1.27, 5.52)		4.07 (1.46, 5.54)		0.235	
**All-cause death**					<0.001	
No	1359	48.92%	755	54.36%		
Yes	1419	51.08%	634	45.64%		

PET–CT, positron emission tomography–computed tomography; AJCC, American Joint Committee on Cancer; CCI, Charlson comorbidity index; EBRT, external beam radiotherapy; RT, radiotherapy; CCRT, concurrent chemoradiotherapy; SD, standard deviation; IQR, interquartile range; y, years; SMD, standardized mean difference.

### Predictors of survival

The findings of univariable and multivariable analyses revealed an association between pretreatment PET–CT and improved survival for the patients with cervical cancer (in the adjusted model, the adjusted hazard ratio [aHR] was 0.88; 95% CI, 0.80–0.97: *P* = 0.010; **Table 2**). Moreover, the results indicated that known prognostic factors, namely age > 60 years (*P* = 0.014); advanced clinical stages of IIA (*P* = 0.026), IIB (*P* = 0.002), III (*P* < 0.001), and IVA (*P* < 0.001); RT alone (*P* < 0.001); adenocarcinoma (*P* < 0.001); CCI score ≥ 1 (*P* < 0.001); and no ICBT (*P* < 0.001), were associated with poor OS (**Table 2**).

**Table 2:** Cox proportional hazard regression analysis of the risk of all-cause death in propensity score–matched patients with cervical cancer.

	Crude HR (95% CI)		*P* value	Adjusted HR (95% CI)		*P* value
Pretreatment PET–CT (ref. = No)						
Yes	0.88	(0.80, 0.96)	0.005	0.88	(0.80, 0.97)	0.010
Age (ref. Age ≤ 40 y)						
40 y < Age ≤ 50 y	1.02	(0.81, 1.15)	0.211	1.07	(0.76, 1.09)	0.233
50 y < Age ≤ 60 y	1.04	(0.81, 1.08)	0.376	1.08	(0.76, 1.12)	0.184
Age > 60 y	1.71	(1.51, 1.94)	<0.001	1.12	(1.07, 1.30)	0.014
CCI score (ref. = 0)						
≥1	1.65	(1.51, 1.82)	<0.001	1.37	(1.24, 1.52)	<0.001
Years of diagnosis (ref. = 2008–2010)						
2011–2014	0.96	(0.91, 1.24)	0.1157	0.99	(0.91, 1.18)	0.6025
2015–2018	0.97	(0.94, 1.21)	0.3046	0.98	(0.84, 1.12)	0.7025
AJCC stages (ref. = Stage IB)						
IIA	1.05	(1.01, 1.54)	0.030	1.39	(1.04, 1.86)	0.026
IIB	1.09	(1.04, 1.27)	0.046	1.38	(1.12, 1.70)	0.002
III	1.51	(1.25, 1.83)	<0.001	2.04	(1.68, 2.48)	<0.001
IVA	4.21	(3.50, 5.05)	<0.001	4.03	(3.32, 4.89)	<0.001
Treatment (ref. = CCRT)						
RT alone	2.51	(2.28, 2.77)	<0.001	1.91	(1.69, 2.16)	<0.001
Differentiation (ref. = Grade I)						
Grade II	1.01	(0.66, 1.28)	0.623	1.20	(0.85, 1.67)	0.297
Grade III	1.00	(0.72, 1.4)	0.986	1.12	(0.8, 1.57)	0.502
Grade IV	1.61	(0.91, 2.54)	0.343	1.38	(0.87, 2.21)	0.170
Histological type (ref. = Squamous cell carcinoma)						
Adenocarcinoma	1.77	(1.55, 2.01)	<0.001	1.75	(1.53, 2)	<0.001
Medical center (ref. = Nonmedical centers)						
Yes	0.89	(0.81, 1.97)	0.279	0.95	(0.86, 1.05)	0.3251
Brachytherapy (ref. = No brachytherapy)						
Yes	0.24	(0.22, 0.26)	<0.001	0.42	(0.38, 0.46)	<0.001

PET–CT, positron emission tomography–computed tomography; HR, hazards ratio; aHR, adjusted hazard ratio; CI, confidence interval; AJCC, American Joint Committee on Cancer; CCI, Charlson comorbidity index; RT, radiotherapy; CCRT, concurrent chemoradiotherapy; y, years.

*All covariates mentioned in **Table 2** were adjusted.

### Stratified analysis of the effect of pretreatment PET–CT

**Figure 1:**
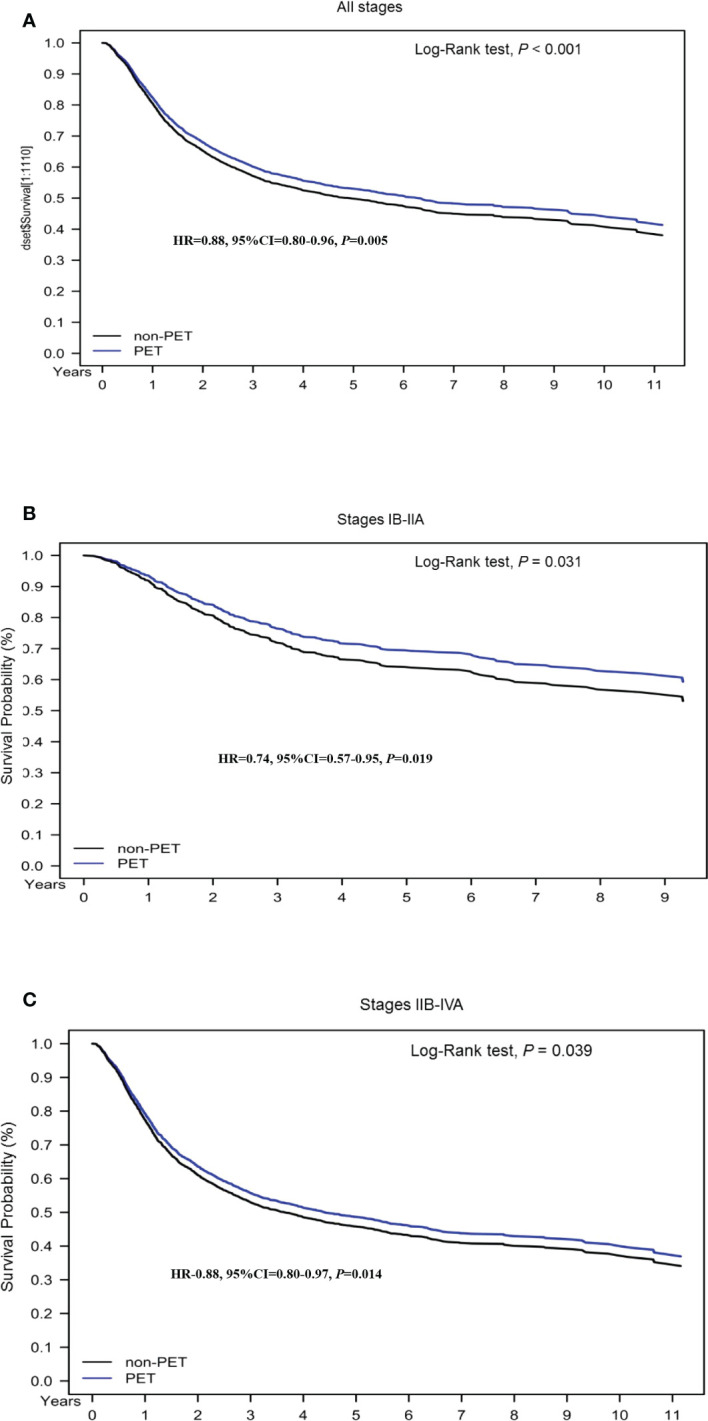
Kaplan–Meier overall survival curves for propensity score–matched patients with cervical cancer. **(A)** All stages; **(B)** stage IB–IIA; **(C)** stage IIB–IVA.

To determine the effect of pretreatment PET–CT on various AJCC clinical stages, we stratified the stages (IB–IIA and III–IVA) by using a Cox regression model after adjustment for age, histology, tumor differentiation, AJCC clinical stages, treatments, cumulative EBRT dose, cumulative platinum dose, cumulative ICBT dose, CCI scores, diagnosis year, and hospital levels (**Tables 3**, **4**). The prognostic factors were similar to those determined in the nonstage stratification analysis. Regardless of the cancer stage (early or advanced), pretreatment PET–CT was significantly superior to nonpretreatment PET–CT in terms of all-cause death (aHR, 0.78; 95% CI, 0.60–0.92; *P* = 0.013 and aHR, 0.90; 95% CI, 0.81–0.99; *P* = 0.039 for early and advanced stages, respectively). In the pretreatment and nonpretreatment groups, the 5-year OS was 54.56% and 50.11%, respectively, for all disease stages (*P* < 0.001); 71.87% and 64.92%, respectively, for stage IB–IIA disease (*P* = 0.031); and 50.73% and 46.832, respectively, for stage IIB–IVA disease (*P* = 0.038; **Figures 1A–C**). In both the groups, we noted the association of early- and advanced-stage cervical cancer treated with RT or CCRT with OS.

**Table 3:** Cox proportional hazard regression analysis of the risk of all-cause death in propensity score–matched patients with AJCC stage IB–IIA cervical cancer.

	Crude HR (95% CI)		*P* value	Adjusted HR (95% CI)		*P* value
Pretreatment PET–CT (ref. = No)						
Yes	0.74	(0.57, 0.95)	0.019	0.78	(0.60, 0.92)	0.013
Age (ref. Age ≤ 40 y)						
40 y < Age ≤ 50 y	1.04	(0.74, 1.48)	0.8091	1.05	(0.73, 1.49)	0.8064
50 y < Age ≤ 60 y	1.12	(0.77, 1.63)	0.5591	1.02	(0.69, 1.53)	0.9052
Age > 60 y	2.11	(1.54, 2.88)	<0.001	1.64	(1.12, 2.38)	0.0101
CCI score(ref. = 0)						
≥1	1.87	(1.48, 2.36)	<0.001	1.61	(1.26, 2.07)	<0.001
Years of diagnosis(ref. = 2008–2010)						
2011–2014	1.20	(0.86, 1.65)	0.281	1.16	(0.82, 1.65)	0.393
2015–2018	1.20	(0.85, 1.68)	0.301	1.02	(0.7, 1.50)	0.913
AJCC stage(ref. = Stage IB)						
IIA	1.16	(0.86, 1.56)	0.334	1.39	(1.01, 1.89)	0.040
Treatment(ref. = CCRT)						
RT alone	1.07	(0.56, 1.49)	0.373	1.04	(0.58, 1.11)	0.228
Differentiation(ref. = Grade I)						
Grade II	1.36	(0.50, 3.69)	0.543	2.81	(0.99, 7.97)	0.154
Grade III	1.37	(0.50, 3.76)	0.535	2.25	(0.79, 6.39)	0.129
Grade IV	2.07	(0.52, 8.29)	0.303	2.65	(0.64, 11.00)	0.179
Histological type(ref. = Squamous cell carcinoma)						
Adenocarcinoma	1.60	(1.17, 2.18)	0.0033	1.86	(1.34, 2.6)	0.0002
**Medical cente**r (ref. = No)						
Yes	0.76	(0.61, 1.15)	0.2182	0.77	(0.59, 1.19)	0.2439
Brachytherapy (ref. = No)						
Yes	0.22	(0.17, 0.27)	<0.001	0.31	(0.24, 0.4)	<0.001

PET–CT, positron emission tomography–computed tomography; HR, hazard ratio; aHR, adjusted hazard ratio; CI, confidence interval; AJCC, American Joint Committee on Cancer; CCI, Charlson comorbidity index; RT, radiotherapy; CCRT, concurrent chemoradiotherapy; y, years.

*All covariates mentioned in **Table 2** were adjusted.

**Table 4:** Cox proportional hazard regression analysis of the risk of all-cause death in propensity score–matched patients with AJCC stage IIB–IVA cervical cancer.

	Crude HR (95% CI)		*P* value	Adjusted HR (95% CI)		*P* value
Pretreatment PET–CT (ref. = No)						
Yes	0.88	(0.80, 0.97)	0.014	0.90	(0.81, 0.99)	0.039
Age (ref. Age ≤ 40 y)						
40 y < Age ≤ 50 y	1.06	(0.75, 1.11)	0.054	1.03	(0.72, 1.06)	0.113
50 y < Age ≤ 60 y	0.88	(0.75, 1.03)	0.104	1.04	(0.72, 1.09)	0.135
Age > 60 y	1.65	(1.44, 1.89)	<0.001	1.04	(1.01, 1.22)	0.044
CCI score (ref. = 0)						
≥1	1.65	(1.49, 1.83)	<0.001	1.33	(1.19, 1.48)	<0.001
Years of Diagnosis (ref. = 2008–2010)						
2011–2014	1.12	(0.99, 1.27)	0.082	1.02	(0.89, 1.18)	0.747
2015–2018	1.08	(0.95, 1.23)	0.258	0.96	(0.82, 1.11)	0.562
AJCC stage (ref. = Stage IIB)						
III	1.16	(1.03, 2.93)	0.015	1.14	(1.09, 2.88)	0.036
IVA	2.67	(1.11, 6.43)	0.028	3.00	(1.24, 7.26)	0.014
Treatment (ref. = CCRT)						
RT alone	3.11	(2.79, 3.47)	<0.001	2.02	(1.76, 2.31)	<0.001
Differentiation (ref. = Grade I)						
Grade II	0.87	(0.61, 1.23)	0.432	1.10	(0.77, 1.57)	0.590
Grade III	0.93	(0.65, 1.32)	0.667	1.05	(0.73, 1.49)	0.809
Grade IV	1.55	(0.95, 2.52)	0.078	1.33	(0.81, 2.18)	0.265
Histological type (ref. = Squamous cell carcinoma)						
Adenocarcinoma	1.89	(1.64, 2.17)	<0.001	1.77	(1.52, 2.05)	<0.001
Medical center (ref. = Nonmedical centers)						
Medical centers	0.92	(0.84, 1.02)	0.109	0.99	(0.89, 1.11)	0.909
Brachytherapy (ref. = No)						
Yes	0.26	(0.24, 0.29)	<0.001	0.45	(0.4, 0.5)	<0.001

PET–CT, positron emission tomography–computed tomography; HR, hazard ratio; aHR, adjusted hazard ratio; CI, confidence interval; AJCC, American Joint Committee on Cancer; CCI, Charlson comorbidity index; RT, radiotherapy; CCRT, concurrent chemoradiotherapy; y, years.

*All covariates mentioned in **Table 2** were adjusted.

## Discussion

Cervical cancer may metastasize to the pelvic or paraaortic lymph nodes as well as more distal nodes (14). Lymph node involvement is associated with poor prognosis and affects decisions regarding the design of RT fields (14). Whole-body FDG-PET–CT, in which both PET and CT are performed using an integrated PET–CT scanner, is the preferred imaging modality for detecting lymph node metastases (5, 6, 15). If the technology is not available, lymph nodes are evaluated through abdominopelvic CT with contrast (2). Pelvic MRI without or with contrast is another second-line alternative because its diagnostic performance is comparable to that of CT (2, 5, 6, 15). All women with cervical cancer should undergo a lymph node evaluation for appropriate staging and treatment (16, 17). For women with cervical cancer (stage IB–IVA) for whom primary RT or CCRT is planned, imaging prior to RT can be performed to evaluate the disease extent, with particular focus on lymph node metastases, to provide information necessary for designing RT fields (18, 19). A meta-analysis of 72 studies including 5042 patients with cervical cancer reported the following sensitivity and specificity values for the detection of lymph node metastasis: PET (75% and 98%), MRI (56% and 93%), and CT (58% and 92%), respectively (5). The more accurate detection of lymph nodes results in a more accurate RT field, thereby possibly contributing to OS benefits. However, studies have reported conflicting results regarding the association between OS and pretreatment PET in patients with cervical cancer (20–23). Only one short-term follow-up randomized controlled trial (RCT) including a small sample size and using conventional 2D-RT reported that pretreatment PET (instead of PET–CT) improved the detection of pelvic metastasis or paraaortic lymph nodes in patients with cervical cancer with pelvic lymph node positivity on pelvic MRI (AJCC stage III at least); however, the improved detection may not translate into a survival benefit (23). No study with an adequate sample size and a long-term follow-up and using a head-to-head PSM design mimicking the RCT has examined the survival benefit of pretreatment PET–CT in patients with stage IB–IVA cervical cancer. In the current study including the largest sample size and a long-term follow-up and using modern RT techniques and PET facilities integrated with the CT scan, pretreatment PET–CT was significantly superior to nonpretreatment PET–CT in terms of all-cause death (aHR, 0.78; 95% CI, 0.60–0.92; *P* = 0.013 and aHR, 0.90; 95% CI, 0.81–0.99; *P* = 0.039 for early and advanced stages, respectively; **Table 3**).

This is the largest study using the head-to-head PSM design to balance potential confounding factors associated with mortality in patients with cervical cancer receiving IG-IMRT with or without pretreatment PET–CT. After PSM, all cofounding factors were balanced (**Table 1**). We believe that the selection bias would be minimal between the case and control groups. The results of multivariable Cox regression model analysis revealed that age > 60 years; advanced clinical stages of IIA, IIB, and IVA; RT alone; adenocarcinoma; CCI score ≥ 1; no ICBT; and nonpretreatment PET–CT were associated with poor OS. The poor prognostic factors are consistent with those reported by previous studies (24–27). Our study is the first to report poor prognostic factors for OS in patients with cervical cancer receiving the modern RT technique of IG-IMRT. Old age, advanced stages, RT alone, adenocarcinoma, CCI score ≥ 1, no IBCT, and nonpretreatment PET–CT were determined as poor prognostic factors for OS, even in the era of IG-IMRT. Moreover, although patients with cervical cancer were treated with the contemporary RT technique of IG-IMRT, ICBT was still necessary and IG-IMRT was insufficient as an alternative treatment to ICBT; this finding is consistent with that of a previous study (27).

Uncertainty regarding patient positioning requires clinicians to add extra margins to target volumes beyond those based on original tumor images (28–30). This uncertainty may be due to imprecision in patient positioning used on a daily basis, despite immobilization, or to inherent organ motion (28–30). During each treatment, the real-time imaging of the treatment target and normal organs allows for the minimization of such additional margins and the reduction of irradiated volumes, leading to a decreased risk of missing a target (28–30). This technology is collectively referred to as IGRT and employs various methods for real-time imaging and treatment adjustment. Refinements to 3D-CRT include IMRT and IGRT. Conformal therapy is generally used to reduce toxicity (28–30). The reduction in toxicity has enabled performing dose escalation trials for improving long-term tumor control or OS. Tsai et al. performed a RCT by including a small sample size and a short-term follow-up and demonstrated that pretreatment PET did not result in survival benefits but only enhanced the detection of extrapelvic metastasis, mainly paraaortic lymph nodes (23). These findings might be attributed to the use of old RT techniques (2D-RT) that resulted in more toxicity or insufficient irradiation doses to extrapelvic metastasis or paraaortic lymph nodes (23). However, compared with old RT techniques, such as 2D-RT, the dose escalation in IG-IMRT might result in less toxicity and more precision, leading to the accurate delineation of cervical cancer (31). Therefore, pretreatment PET–CT might be associated with longer OS compared with nonpretreatment PET–CT in the era of IG-IMRT.

In the future, a large and well-designed RCT may be necessary to confirm the survival benefit of pretreatment PET–CT for patients with stage IB–IVA cervical cancer receiving RT or CCRT in the era of IG-IMRT, although the inclusion of a control arm (nonpretreatment PET–CT) for patients with cervical cancer, especially for those with advanced stages, can be an ethical problem because of the accurate detection of extrapelvic metastasis or paraaortic lymph nodes (23). Owing to the difficulty in performing this type of RCT, a large retrospective observational study might be necessary. However, in a large cohort, with in-depth postprocessing such as PSM, a retrospective study of an existing database without randomization cannot mimic a RCT, possibly resulting in a selection bias. Thus, a study with the PSM design can be performed to address the question regarding the use of available data and complement the lack of well-designed RCTs.

Our study strengths are as follows. This is the first, largest, long-term follow-up cohort study using a homogenous modality with integrated PET–CT to estimate the survival outcomes of pretreatment ^18^This study investigated the effects of FDG-PET–CT or nonpretreatment PET–CT on patients with cervical cancer stratified by different clinical stages. Comparative reports for different clinical stages, sufficient sample sizes, long-term follow-up periods, homogenous ^18^FDG-PET–CT modalities, and PSM design mimicking the RCT are lacking. In the present study, pretreatment ^18^FDG-PET–CT was associated with survival benefits for the patients with stage IB–IVA cervical cancer. Our result suggests that pretreatment ^18^FDG-PET–CT is necessary for patients with cervical cancer receiving RT or CCRT, especially in the era of IG-IMRT. Our findings should be considered in future clinical practice and prospective clinical trials.

This study has some limitations. First, because all patients with cervical cancer were enrolled from the Asian population, the corresponding ethnic susceptibility compared with the non-Asian population remains unclear; hence, our results should be cautiously extrapolated to non-Asian populations. However, no evidence demonstrates differences in survival outcomes between Asian and non-Asian patients with cervical cancer receiving RT or CCRT. Second, although the main advantage of PSM is that it enables a more precise estimation of the intervention response, PSM cannot control for factors not accounted for in the model and is based on an explicit selection bias toward patients who could be matched (i.e., those who could not be matched are not part of the scope of inference). Third, we do not have data for young cervical cancer patients who may require fertility-sparing treatment, and this may limit the generalizability of our findings to this population. Fourth, the diagnoses of all comorbidities were based on *ICD-9-CM* or *ICD-10-CM* codes. However, the combination of the TCR and NHIRD appears to be a valid resource for population studies on comorbidities (32–34). Moreover, the TCR administration randomly reviews charts and interviews patients to verify the accuracy of diagnoses, and hospitals with outlier chargers or practices may be audited and subsequently heavily penalized if malpractice or discrepancies are identified. Despite these limitations, a major strength of the present study is the use of a nationwide population-based registry with detailed baseline and treatment information. Lifelong follow-up was possible through the linkage of the registry with the national Cause of Death database. Considering the magnitude and statistical significance of the observed effects in the current study, the limitations are unlikely to affect our conclusions.

## Conclusion

Pretreatment ^18^FDG-PET–CT might be associated with longer survival in patients with stage IB–IVA cervical cancer receiving RT or CCRT, especially in the era of IG-IMRT.

## Data availability statement

The raw data supporting the conclusions of this article will be made available by the authors, without undue reservation.

## Ethics statement

The study protocols were reviewed and approved by the Institutional Review Board of Tzu-Chi Medical Foundation (IRB109-015-B). Written informed consent for participation was not required for this study in accordance with the national legislation and the institutional requirements.

## Author contributions

Conception and design, W-MC, MC, B-CS, and S-YW. Financial support, Lo-Hsu Medical Foundation, LotungPoh-Ai Hospital, supports S-YW’s work (Funding Number: 11001, 11010, 11013 and 11103). Collection and assembly of data, W-MC, B-CS, and S-YW. Data analysis and interpretation, W-MC, B-CS, and S-YW. Administrative support, S-YW. Manuscript writing, W-MC. Final approval of manuscript, all authors. All authors contributed to the article and approved the submitted version.

## References

[1] Sung H Ferlay J Siegel RL Laversanne M Soerjomataram I Jemal A. Global cancer statistics 2020: GLOBOCAN estimates of incidence and mortality worldwide for 36 cancers in 185 countries. CA Cancer J Clin (2021) 71:209–49. doi: 10.3322/caac.21660 33538338

[2] Oncology N.C.P.G.I. NCCN clinical practice guidelines in oncology: Cervical cancer (2021). 94 N Woodhull Rd, Huntington, NY 11743: Harborside Press, LLC (Accessed 10/26/2021).

[3] Narayanan P Sahdev A. The role of (18)F-FDG PET CT in common gynaecological malignancies. Br J Radiol (2017) 90:20170283. doi: 10.1259/bjr.20170283 28830238 PMC5963379

[4] Gee MS Atri M Bandos AI Mannel RS Gold MA Lee SI. Identification of distant metastatic disease in uterine cervical and endometrial cancers with FDG PET/CT: Analysis from the ACRIN 6671/GOG 0233 multicenter trial. Radiology (2018) 287:176–84. doi: 10.1148/radiol.2017170963 PMC5881639 29185901

[5] Selman TJ Mann C Zamora J Appleyard TL Khan K. Diagnostic accuracy of tests for lymph node status in primary cervical cancer: A systematic review and meta-analysis. CMAJ (2008) 178:855–62. doi: 10.1503/cmaj.071124 PMC2267838 18362381

[6] Atri M Zhang Z Dehdashti F Lee SI Ali S Marques H. Utility of PET-CT to evaluate retroperitoneal lymph node metastasis in advanced cervical cancer: Results of ACRIN6671/GOG0233 trial. Gynecol Oncol (2016) 142:413–9. doi: 10.1016/j.ygyno.2016.05.002 PMC4993667 27178725

[7] Leblanc E Gauthier H Querleu D Ferron G Zerdoud S Morice P. Accuracy of 18-fluoro-2-deoxy-D-glucose positron emission tomography in the pretherapeutic detection of occult para-aortic node involvement in patients with a locally advanced cervical carcinoma. Ann Surg Oncol (2011) 18:2302–9. doi: 10.1245/s10434-011-1583-9 21347790

[8] Ramirez PT Jhingran A Macapinlac HA Euscher ED Munsell MF Coleman RL. Laparoscopic extraperitoneal para-aortic lymphadenectomy in locally advanced cervical cancer: A prospective correlation of surgical findings with positron emission tomography/computed tomography findings. Cancer (2011) 117:1928–34. doi: 10.1002/cncr.25739 PMC4286384 21509770

[9] Chang CL Yuan KS Wu SY. High-dose or low-dose cisplatin concurrent with radiotherapy in locally advanced head and neck squamous cell cancer. Head Neck (2017) 39:1364–70. doi: 10.1002/hed.24763 28370614

[10] Lin KC Chen TM Yuan KS Wu ATH Wu SY. Assessment of predictive scoring system for 90-day mortality among patients with locally advanced head and neck squamous cell carcinoma who have completed concurrent chemoradiotherapy. JAMA Netw Open (2020) 3:e1920671. doi: 10.1001/jamanetworkopen.2019.20671 32215631 PMC12507455

[11] Liu WC Liu HE Kao YW Qin L Lin KC Fang CY. Definitive intensity-modulated radiotherapy or surgery for early oral cavity squamous cell carcinoma: Propensity-score-matched, nationwide, population-based cohort study. Head Neck (2020) 43:1142–52. doi: 10.1016/j.radonc.2020.08.016 33314548

[12] Zhang Z Kim HJ Lonjon G Zhu Y Written on Behalf Of, A.M.E.B.-D.C.T.C.G Balance diagnostics after propensity score matchingBAnn Transl Med (2019) 7:16. doi: 10.21037/atm.2018.12.10 30788363 PMC6351359

[13] Austin PC. Optimal caliper widths for propensity-score matching when estimating differences in means and differences in proportions in observational studies. Pharm Stat (2011) 10:150–61. doi: 10.1002/pst.433 PMC3120982 20925139

[14] Singh N Arif S. Histopathologic parameters of prognosis in cervical cancer–a review. Int J Gynecol Cancer (2004) 14:741–50. doi: 10.1111/j.1048-891X.2004.014504.x 15361180

[15] Sironi S Buda A Picchio M Perego P Moreni R Pellegrino A. Lymph node metastasis in patients with clinical early-stage cervical cancer: detection with integrated FDG PET/CT. Radiology (2006) 238:272–9. doi: 10.1148/radiol.2381041799 16304090

[16] Bhatla N Berek JS Cuello Fredes M Denny LA Grenman S Karunaratne K. Revised FIGO staging for carcinoma of the cervix uteri. Int J Gynaecol. Obstet. (2019) 145:129–35. doi: 10.1002/ijgo.12749 30656645

[17] Olawaiye AB Baker TP Washington MK Mutch DG. The new (Version 9) American joint committee on cancer tumor, node, metastasis staging for cervical cancer. CA Cancer J Clin (2021) 71:287–98. doi: 10.3322/caac.21663 33784415

[18] Varia MA Bundy BN Deppe G Mannel R Averette HE Rose PG. Cervical carcinoma metastatic to para-aortic nodes: extended field radiation therapy with concomitant 5-fluorouracil and cisplatin chemotherapy: A gynecologic oncology group study. Int J Radiat Oncol Biol Phys (1998) 42:1015–23. doi: 10.1016/S0360-3016(98)00267-3 9869224

[19] Du XL Sheng XG Jiang T Yu H Yan YF Gao R. Intensity-modulated radiation therapy versus para-aortic field radiotherapy to treat para-aortic lymph node metastasis in cervical cancer: Prospective study. Croat. Med J (2010) 51:229–36. doi: 10.3325/cmj.2010.51.229 PMC2897095 20564766

[20] Grigsby PW Siegel BA Dehdashti F. Lymph node staging by positron emission tomography in patients with carcinoma of the cervix. J Clin Oncol (2001) 19:3745–9. doi: 10.1200/JCO.2001.19.17.3745 11533097

[21] Kidd EA Siegel BA Dehdashti F Grigsby PW. Pelvic lymph node f-18 fluorodeoxyglucose uptake as a prognostic biomarker in newly diagnosed patients with locally advanced cervical cancer. Cancer (2010) 116:1469–75. doi: 10.1002/cncr.24972 20108309

[22] Kidd EA Siegel BA Dehdashti F Rader JS Mutch DG Powell MA. Lymph node staging by positron emission tomography in cervical cancer: relationship to prognosis. J Clin Oncol (2010) 28:2108–13. doi: 10.1200/JCO.2009.25.4151 20308664

[23] Tsai CS Lai CH Chang TC Yen TC Ng KK Hsueh S. A prospective randomized trial to study the impact of pretreatment FDG-PET for cervical cancer patients with MRI-detected positive pelvic but negative para-aortic lymphadenopathy. Int J Radiat Oncol Biol Phys (2010) 76:477–84. doi: 10.1016/j.ijrobp.2009.02.020 19464824

[24] Wu S-Y Huang E-Y Lin H. Optimal treatments for cervical adenocarcinoma. Am J Cancer Res (2019) 9:1224–34. PMC6610057 31285954

[25] Zhang J Qin L Chen HM Hsu HC Chuang CC Chen D. Outcome patterns of cervical adenocarcinoma and squamous cell carcinoma following curative surgery: Before and after propensity score matching analysis of a cohort study. Am J Cancer Res (2020) 10:1793–807. PMC7339276 32642291

[26] Zhang J Qin L Chen HM Hsu HC Chuang CC Chen D. Overall survival, locoregional recurrence, and distant metastasis of definitive concurrent chemoradiotherapy for cervical squamous cell carcinoma and adenocarcinoma: before and after propensity score matching analysis of a cohort study. Am J Cancer Res (2020) 10:1808–20. PMC7339279 32642292

[27] Zhang J Sun M Li N Miao M Yang Y Hsu HC. Contemporary external beam radiotherapy boost or high dose-rate brachytherapy boost for cervical cancer: a propensity-score-matched, nationwide, population-based cohort study. Am J Cancer Res (2021) 11:1719–32. PMC8085872 33948385

[28] Guerrero Urbano MT Nutting CM. Clinical use of intensity-modulated radiotherapy: part I. Br J Radiol (2004) 77:88–96. doi: 10.1259/bjr/84246820 15010378

[29] Pow EH Kwong DL Mcmillan AS Wong MC Sham JS Leung LH. Xerostomia and quality of life after intensity-modulated radiotherapy vs. conventional radiotherapy for early-stage nasopharyngeal carcinoma: initial report on a randomized controlled clinical trial. Int J Radiat Oncol Biol Phys (2006) 66:981–91. doi: 10.1016/j.ijrobp.2006.06.013 17145528

[30] Garden AS Morrison WH Wong PF Tung SS Rosenthal DI Dong L. Disease-control rates following intensity-modulated radiation therapy for small primary oropharyngeal carcinoma. Int J Radiat Oncol Biol Phys (2007) 67:438–44. doi: 10.1016/j.ijrobp.2006.08.078 PMC4125020 17141972

[31] Chang CL Tsai HC Lin WC Chang JH Hsu HL Chow JM. Dose escalation intensity-modulated radiotherapy-based concurrent chemoradiotherapy is effective for advanced-stage thoracic esophageal squamous cell carcinoma. Radiother Oncol (2017) 125:73–9. doi: 10.1016/j.radonc.2017.08.025 28923576

[32] Lin CC Lai MS Syu CY Chang SC Tseng FY. Accuracy of diabetes diagnosis in health insurance claims data in Taiwan. J Formos. Med Assoc (2005) 104:157–63. doi: 10.1002/pds.2087 15818428

[33] Cheng CL Kao YH Lin SJ Lee CH Lai ML. Validation of the national health insurance research database with ischemic stroke cases in Taiwan. Pharmacoepidemiol Drug Saf (2011) 20:236–42. doi: 10.2188/jea.je20140076 21351304

[34] SEER cancer stat facts: Cervix uteri cancer (2017). Available at: https://seer.cancer.gov/statfacts/html/cervix.html (Accessed February 11, 2017).

